# Starch phosphorylation in potato tubers is influenced by allelic variation in the genes encoding glucan water dikinase, starch branching enzymes I and II, and starch synthase III

**DOI:** 10.3389/fpls.2015.00143

**Published:** 2015-03-10

**Authors:** Margaret A. Carpenter, Nigel I. Joyce, Russell A. Genet, Rebecca D. Cooper, Sarah R. Murray, Alasdair D. Noble, Ruth C. Butler, Gail M. Timmerman-Vaughan

**Affiliations:** ^1^The New Zealand Institute for Plant and Food Research Ltd.Lincoln, New Zealand; ^2^The New Zealand Institute for Plant and Food Research Ltd.Auckland, New Zealand; ^3^AgResearchLincoln, New Zealand

**Keywords:** starch, phosphorylation, potato, glucan water dikinase, starch branching enzyme, starch synthase

## Abstract

Starch phosphorylation is an important aspect of plant metabolism due to its role in starch degradation. Moreover, the degree of phosphorylation of starch determines its physicochemical properties and is therefore relevant for industrial uses of starch. Currently, starch is chemically phosphorylated to increase viscosity and paste stability. Potato cultivars with elevated starch phosphorylation would make this process unnecessary, thereby bestowing economic and environmental benefits. Starch phosphorylation is a complex trait which has been previously shown by antisense gene repression to be influenced by a number of genes including those involved in starch synthesis and degradation. We have used an association mapping approach to discover genetic markers associated with the degree of starch phosphorylation. A diverse collection of 193 potato lines was grown in replicated field trials, and the levels of starch phosphorylation at the C6 and C3 positions of the glucosyl residues were determined by mass spectrometry of hydrolyzed starch from tubers. In addition, the potato lines were genotyped by amplicon sequencing and microsatellite analysis, focusing on candidate genes known to be involved in starch synthesis. As potato is an autotetraploid, genotyping included determination of allele dosage. Significant associations (*p* < 0.001) were found with SNPs in the glucan water dikinase (*GWD*), starch branching enzyme I (*SBEI*) and the starch synthase III (*SSIII*) genes, and with a SSR allele in the *SBEII* gene. SNPs in the *GWD* gene were associated with C6 phosphorylation, whereas polymorphisms in the *SBEI* and *SBEII* genes were associated with both C6 and C3 phosphorylation and the SNP in the *SSIII* gene was associated with C3 phosphorylation. These allelic variants have potential as genetic markers for starch phosphorylation in potato.

## Introduction

Starch is the major storage carbohydrate in plants and comprises most of the dry matter in potato tubers. Starch represents the largest source of carbohydrates in human food and has a range of industrial uses. Starch is made up of chains of α-D-glucose units which can be unbranched (amylose) or branched (amylopectin). The native starches of almost all plants are phosphorylated, the extent of which varies between plant species, and is greater in potato starch than in cereal starches. The degree of phosphorylation of starch determines its physicochemical properties and is therefore relevant for industrial uses of starch, which include the manufacturing of paper, adhesives, textiles, and foods (Kraak, 1992). Phosphorylation increases the hydration capacity of starch pastes, thereby influencing peak viscosity, gel-forming capacity, swelling power and paste stability (Vikso-Nielsen et al., 2001; Jobling, 2004). Starch phosphorylation is also an important aspect of plant metabolism due to its role in starch degradation (Lorberth et al., 1998). Phosphorylation occurs only in the amylopectin fraction of starch. The glucosyl residues that comprise amylopectin can be phosphorylated at either the C6 or C3 position (Tabata and Hizukuri, 1971). In potato tuber starch, C6 phosphorylation predominates, making up 70–80% of the phosphorylation, while C3 makes up 20–30% (Bay-Smidt et al., 1994; Haebel et al., 2008; Carpenter et al., 2012).

A number of genes have been shown by genetic modification experiments to have a role in starch phosphorylation in potato. The *GWD* (or *R1*) gene encodes the protein glucan/water dikinase (GWD) which catalyzes the addition of phosphate groups to the C6 position of the glycosyl residues (Vikso-Nielsen et al., 2001). Down-regulation of the *GWD* gene resulted in decreased C6 phosphorylation of potato starch (Lorberth et al., 1998; Vikso-Nielsen et al., 2001; Wickramasinghe, 2009). Potato also has a *PWD* gene which encodes phosphoglucan/water dikinase (PWD) (Orzechowski et al., 2012). Down-regulation of *PWD* reduced C3 phosphorylation in *Arabidopsis* (Ritte et al., 2006) showing that PWD adds phosphate at the C3 position. C3 phosphorylation is dependent on prior C6 phosphorylation by GWD (Baunsgaard et al., 2005). Starch phosphorylation is also influenced by the activity of the genes encoding starch branching enzymes. Antisense inhibition of the genes encoding the starch branching enzymes SBEI and SBEII, either singly or together, resulted in an increase in the phosphorus content of the starch (Safford et al., 1998; Schwall et al., 2000) and similarly an increase in the C6 phosphorylation (Wischmann et al., 2005; Wickramasinghe, 2009). This was accompanied by a decrease in the amylopectin content and an increase in the length of amylopectin branches (Schwall et al., 2000; Wischmann et al., 2005; Wickramasinghe, 2009). There is also evidence that the starch synthase genes may influence starch phosphorylation in potato. Suppression of the granule-bound starch synthase gene (*GBSS*) caused a small increase in C6 phosphorylation and an increase in amylopectin content (Kozlov et al., 2007), while reduction in the activity of starch synthase II (SSII) caused a 40% reduction in C6 phosphorylation (Kossmann et al., 1999). Antisense suppression of the starch synthase I gene (*SSI*) had no significant effect on starch phosphorylation (Kossmann et al., 1999). Additionally, reduction in the activity of starch synthases II and/or III (SSII, SSIII) resulted in changes to gelatinization behavior and amylopectin structure of the starch, characteristics which are associated with starch phosphorylation (Edwards et al., 1999). In this case the phosphorylation was not determined. Several of these genes have also been associated with the degree of starch phosphorylation in potato through candidate gene mapping approaches, specifically the *GWD, SSII*, and *SSIII* genes (Uitdewilligen, 2012; Werij et al., 2012).

Association mapping is an increasingly popular approach for identifying loci linked to traits of commercial interest in plants (D'hoop et al., 2008). This approach has been used in potatoes for a variety of traits such as cold-induced sweetening (Baldwin et al., 2011), disease resistance (Simko et al., 2004; Pajerowska-Mukhtar et al., 2009), tuber bruising and enzymatic discoloration (Urbany et al., 2011) and other tuber characteristics (D'hoop et al., 2008; Li et al., 2008; Schreiber et al., 2014). Association mapping employs collections of existing germplasm, thus avoiding the need to select and cross parents, and propagate the offspring. If a suitably diverse collection is used, the gene-trait associations which are identified will be applicable over a broad range of cultivars, and may identify desirable alleles which are not present in current breeding programs. Association mapping can also confer a higher resolution than QTL mapping, depending on the extent of linkage disequilibrium, (Flint-Garcia et al., 2003; Gaut and Long, 2003) such that it works well with a candidate gene approach, where genotyping focuses on genes that are known or suspected to be associated with the trait of interest. Association mapping can be confounded by population structure or kinship within the collection, generating false positive results (Flint-Garcia et al., 2003). Therefore, the relatedness of individuals within the collection needs to be determined and the population structure and kinship taken into account (Yu et al., 2006; Stich and Melchinger, 2009), both of which add complexity to the analysis.

The potato (*Solanum tuberosum*) is a highly heterozygous, autotetraploid, outcrossing species which is propagated clonally. The breeding history of the cultivated potato has created a situation in which many current cultivars from around the world are related by a complex network of relationships. Genes from wild species (*S. vernei, S. demissum*) have been introduced during the introgression of disease resistance genes to try to overcome disease problems in commercial cultivars (Pajerowska-Mukhtar et al., 2009). Traditional lines of uncertain origin also exist. Autotetraploidy adds complexity to genetic analysis as many more combinations of alleles are possible than in a diploid. For example, a biallelic locus can have up to five different genotypes, two homozygous and three heterozygous, depending on the number of copies of each allele. The number of copies of each allele can affect the phenotype in an additive or dominant fashion, or somewhere between (Gallais, 2003). Therefore, the allele dosage is an important aspect of the data and should, if possible, be included.

Here, we report an association mapping analysis of starch phosphorylation in potato in which mass spectrometry was used to determine the C6 and C3 phosphorylation of tuber starch from two field trials, and genotyping was focused on eight candidate genes. The association analysis was performed using a mixed model which included population structure and kinship estimates derived from molecular markers. This study differs from previous reports which identified genetic markers associated with potato starch phosphorylation (Uitdewilligen, 2012; Werij et al., 2012) in that we have measured both C3 and C6 phosphorylation separately and have chosen a different selection of candidate genes. Our results validate some of the previous reports and also provide new insights.

## Materials and methods

### Plant material

A diverse collection of 193 tetraploid potato lines (mainly *Solanum tuberosum* but including lines with introgression from other species) was grown in field trials at Lincoln, Canterbury, New Zealand, during two consecutive seasons (2011 and 2012). Tubers were planted in October and harvested in March, and the plants were irrigated as required. Climate data for the growing seasons were obtained from the National Climate Database maintened by NIWA (National Institute of Water and Atmospheric Research), New Zealand. The lines and their origins are listed in Supplementary Data 1.

Two replicate plots of each of 192 test lines were planted as well as 48 plots of a control line (“Red Rascal”). Each plot consisted of six plants, planted in two adjacent rows. The 432 plots were planted in a 72 × 6 plot array, with a full replicate (including 24 controls) in each of two adjacent blocks. The position of test lines within the layout was derived from a resolvable block design with blocks of eight, created with CycDesign (Cycsoftware, 2009). Control plots were added to this base design, such that each column of plots (six columns) contained eight control plots, and each set of 9rows of plots (eight sets; 72 rows in all) contained six controls, with a maximum of one control per row.

At maturity, a sample of six typical tubers from the two plants in the middle of the plot was harvested for phenotyping. Cylindrical samples were taken through the middle of each tuber using an 11 mm cork borer. The six cores from each plot were pooled and freeze dried. Samples were processed in plot order, in batches of 36 for starch extraction and 72 for phosphorylation measurement.

### Starch phosphorylation assays

Starch was extracted from freeze-dried tuber core samples. Starch samples (1 mg) were hydrolyzed with trifluoroacetic acid and prepared as described previously (Carpenter et al., 2012). The glucose, glucose 6-phosphate and glucose 3-phosphate content of the hydrolysates were determined by mass spectrometry using external standards for quantitation (Carpenter et al., 2012). The glucose content gave an accurate estimation of the amount of starch hydrolyzed, and was therefore used to convert the glucose 6-phosphate and glucose 3-phosphate measurements to C6 and C3 phosphorylation in nmol/mg starch.

### Genotyping

Genomic DNA was isolated from young leaf tissue using a cetyl trimethylammonium bromide (CTAB) method (Timmerman et al., 1993). The potato lines were genotyped using 21 SSR markers for determination of population structure. Markers were developed for the eight candidate genes using a range of methods including amplicon sequencing, SSR analysis, and by amplification of a region containing an insertion/deletion polymorphism.

SSR markers for population structure were amplified by PCR using previously published primers (Milbourne et al., 1998; Feingold et al., 2005) as shown in Supplementary Data 2. These were selected so that there was at least one SSR marker per chromosome, and in most cases two per chromosome. The forward primer of each pair was modified by the addition of a 20 bp tail with the sequence gacgttgtaaaacgacggcc at the 5′ end, enabling the addition during PCR of a fluorescently labeled “tail primer” (gacgttgtaaaacgacg) (Schuelke, 2000) labeled with FAM (6-carboxy-fluorescine), HEX (hexachloro-6-carboxy-fluorescine) or NED (Applied Biosystems, Carlsbad, California, USA). A short 4 bp “pigtail” gttt was added to the 5′ end of the reverse primer to reduce the appearance of stutter bands (Brownstein et al., 1996). Optimal annealing temperatures and MgCl_2_ concentrations for the PCR reactions were determined empirically as the reported conditions did not always produce satisfactory results. The 15 μl PCRs contained 75 mM TrisHCl pH8.8, 20 mM (NH_4_)_2_SO_4_, 0.01% Tween® 20, 0.2 mM each dNTP, 1 μM each primer, 0.02 μM tail primer, 0.75U Taq (Thermo Fisher Scientific, Waltham, MA, USA), approximately 10 ng genomic DNA and 2.5–4.0 mM MgCl_2_ as shown in Supplementary Data 2. The PCR conditions were one cycle of 95°C for 1 min, 40 cycles of 95°C for 30 s, annealing at the temperature shown in Supplementary Data 2 for 30s, 72°C for 1 min, then one cycle of 72°C for 7 min. PCR products from two to three SSRs were combined in groups such that both amplicon sizes and fluorescent labels differed within the group, and separated by capilliary electrophoresis using an ABI PRISM 3130xl Genetic Analyzer (Applied Biosystems, Carlsbad, California, USA) with the GeneScan 400HD ROX size standard (Applied Biosystems, Carlsbad, California, USA).

Four SSR markers in the candidate genes were also characterized: the STGBSS and STWAX-2 primers (Ghislain et al., 2004) are described in Supplementary Data 2 and the novel *SBEII* and *GWD* SSR primers are shown in Supplementary Data 3. PCR of the candidate gene SSRs was performed as described above, except that the PCR for the *GWD* and *SBEII* SSRs used 2.5 mM MgCl_2_ and annealing at 58°C.

The SSR data for candidate genes and for population structure were analyzed using GeneMarker v1.85 (SoftGenetics, State College Pennsylvania, USA) to identify alleles by product size, while allele copy number was estimated by peak area. When four alleles were present, these were scored as a single copy of each. When a single allele was present, this was scored as four copies of that allele. When two alleles were present, this was scored as 2:2, 3:1 or 2:1 depending on the relative peak areas (a ratio of 2:1 inferred that one allele was null). Where three alleles were present, the largest peak was scored as two copies and the other two as a single copy, except where the three peaks were all of a similar area in which case a null allele was assumed and the three alleles were scored as one copy each.

The eight candidate genes were resequenced to provide sequence data for optimal design of primers to be used for genotyping. The candidate genes were amplified as fragments of 4–15 kb from genomic DNA from four potato lines (4164A3, “Brodick,” V201, Vtn62-33-3). The PCR was performed using the Expand long template PCR system (Roche, Basel, Switzerland) according to the manufacturers' instructions, and using the primers shown in Supplementary Data 3. One of the genes, *SBEII*, was amplified in two pieces as it was not possible to amplify the complete 20 kb gene. The products were normalized to ensure equal representation of all genes, then fragmented and used to construct four bar-coded libraries which were sequenced on a Genome Sequencer FLX instrument (Roche, Basel, Switzerland). The resulting sequence reads were aligned to reference sequences using gsMapper software (Roche, Basel, Switzerland), which identified a list of SNPs (Supplementary Data 4). The SNP positions were converted to a GFF file which was imported into Geneious Pro v5.3.6 (Biomatters, Auckland, New Zealand) where primers for amplicon sequencing were designed so as to avoid having SNPs in the primer binding site.

Primers (shown in Supplementary Data 3) were designed to amplify regions of 300–700 bp from the candidate genes for sequencing. The primers were designed using Primer 3 (Koressaar and Remm, 2007) with an optimal annealing temperature of 60°C and using the default parameters. The 15 μl PCRs contained 75 mM TrisHCl pH8.8, 20 mM (NH_4_)_2_SO_4_, 0.01% Tween 20, 2.5 mM MgCl_2_, 0.2 mM each dNTP, 1μM each primer, 0.75U Taq (Thermo Fisher Scientific, Waltham, MA, USA), and approximately 10 ng genomic DNA. The PCR conditions were one cycle of 95°C for 2 min, 40 cycles of 95°C for 30 s, 58°C for 30s, 72°C for 1 min, then one cycle of 72°C for 7 min. Unincorporated primers were removed from the PCR products using exonuclease I and shrimp alkaline phosphatase (Ibrahim et al., 2001). The PCR products were sequenced in both directions using the amplification primers, except in the case of *SBEII* where a different forward primer (Supplementary Data 3) was used for sequencing in order to avoid an insertion/deletion polymorphism. Sanger sequencing was performed using an ABI BigDye terminator cycle sequencing kit and an ABI PRISM 3130xl Genetic Analyzer (Applied Biosystems, Carlsbad, California, USA). SNPs were detected by aligning sequences using Geneious Pro v5.3.6 (Biomatters, Auckland, New Zealand). The SNP allele dosage was estimated using the DAx data analysis and acquisition software (Van Mierlo Software Consultancy, Eindhoven, The Netherlands). Allele dosages were determined separately for both forward and reverse sequences and then the two results were compared. When discrepancies occurred, the result with the higher sequence quality was used.

A region containing an insertion/deletion polymorphism (InDel) located 0.5 kb upstream of the ATG start codon of the *GBSS* gene was amplified using cdf1 and cdf2 primers (van de Wal et al., 2001). PCR conditions were as for amplicon sequencing except that the annealing temperature was 55°C. PCR products were separated by electrophoresis on a 2% agarose gel and visualized by ethidium bromide staining. Relative band intensity of heterozygotes was determined using GelQuantNET software (http://biochemlabsolutions.com/GelQuantNET.html) and used to group the heterozygotes into three genotypes.

### Statistical analysis of phenotypic data

The C6 and C3 phosphorylation (nmol/mg) for each of the two field trials were analyzed separately. For each type of phosphorylation, mean values were calculated which were adjusted to compensate for spatial variation in the field trial and any local trends in processing (such as a tendency to systematically increase or decrease within a run of the mass spectrometer), using the “Red Rascal” control data. The data were analyzed using a mixed model approach, with the model fitted with restricted maximum likelihood (REML, Payne et al., 2011) as implemented in Genstat (Genstat Committee, 2013). The potato line was included as a fixed effect whereas other potentially important effects were included as random effects. For both C6 and C3 phosphorylation, an AR(1) × AR(1) (Verbyla et al., 1999) correlation model was fitted to account for local trends in the data. In addition, random effects were required for the sets of 72 plots (corresponding to the six columns of plots as in the field layout) and also for the batches of 36 plots used for starch extraction. Overall differences between lines were assessed with an F-test, using the Kenward-Roger estimation for the denominator degrees of freedom (Kenward and Roger, 1997).

### Analysis of population structure and kinship

Population structure of the collection of 192 potato lines was explored by analyzing 21 SSR markers using the program STRUCTURE v2.3 (Pritchard et al., 2000). An admixture model was used, with the number of subpopulations, K, set from 1 to 20 for each of three repetitions. For each run, both the burn-in and the iteration number were set to 100,000. The mean and standard deviation of Ln P(D) were calculated and the best estimate of K was determined by the method of Evanno et al. (2005). A Q matrix was generated for the best estimate of K and used in the association analysis. The Q matrix contained, for each potato line, its estimated membership of each subpopulation. A kinship matrix (K) was calculated from the SSR data according to Loiselle et al. (1995) using the software package SPAGeDI v1.3 (Hardy and Vekemans, 2002), with negative kinship values set to zero.

### Association analysis

A mixed model analysis was used to test for association between phosphorylation phenotypes and SNP/SSR/InDel genotypes following the approach of Pajerowska-Mukhtar et al. (2009) Population structure (Q) and kinship (K) are included in the model to decrease the rate of discovery of false positives (Yu et al., 2006). The genotype and subpopulation membership (Q) were incorporated in the model as fixed effects, with genotype as a factor (number of copies of each allele) and subpopulation membership as a variate. Kinship and residual were included as random effects. The C6 and C3 phosphorylation for each of the two field trials were analyzed separately. The analysis was performed using ASReml-R (Butler et al., 2009).

To address the problem of false discovery when multiple tests are performed simultaneously, *q*-values were calculated using the QVALUE package implemented in R with the default range of lambda values and the bootstrap method (Storey and Tibshirani, 2003). The *p*-values from the four trait data sets (C6 and C3, both years) were combined for the q-value calculation.

Amino acid substitutions resulting from the DNA polymorphisms that were found to be significantly associated with starch phosphorylation were analyzed using PROVEAN software (Choi et al., 2012). This predicted whether an amino acid substitution would affect the biological function of a protein, by referring to the variation observed in homologous sequences.

## Results

### Starch phosphorylation

**Figure 1:**
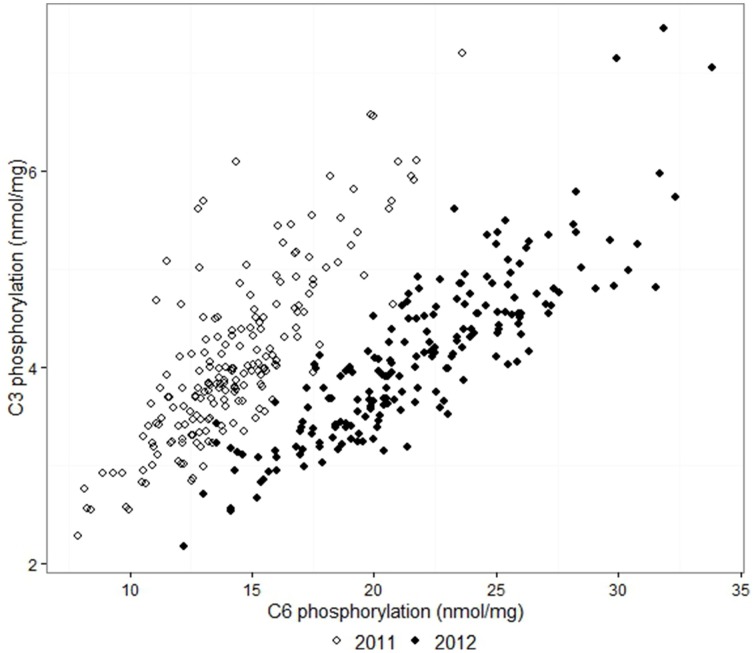
**Comparison between the adjusted means of C3 and C6 phosphorylation from the 2011 and 2012 field trials**. The correlation coefficients *r* were 0.81 and 0.86 respectively.

**Figure 2:**
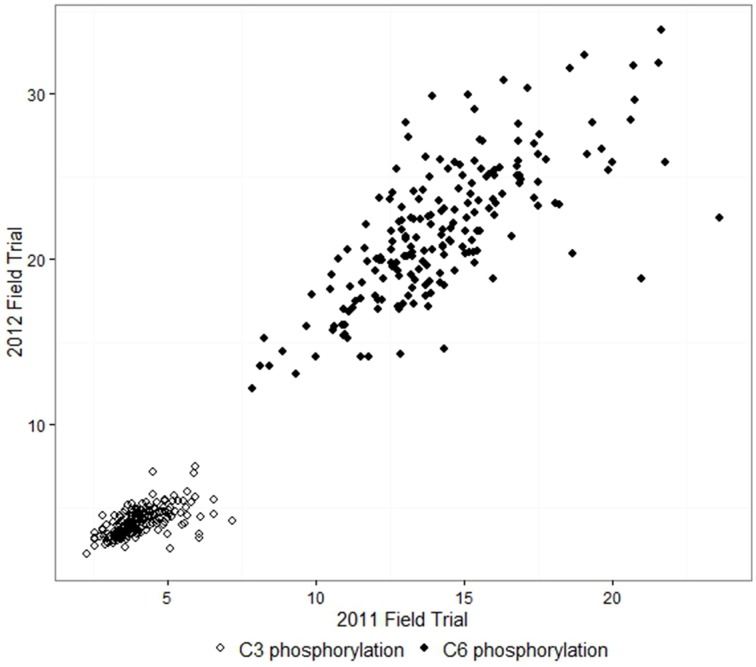
**Comparison between the two field trials: adjusted means of C6 and C3 phosphorylation of starch (nmol/mg) from potato tubers**. The correlation coefficients *r* were 0.72 for C6 and 0.62 for C3.

The adjusted means for C6 phosphorylation varied from 7.9 to 23.6 nmol/mg in samples from the 2011 field trial, and 12.2 to 33.8 nmol/mg in 2012. C3 phosphorylation means were in the range 2.3–7.2 nmol/mg in 2011 and 2.2–7.5 nmol/mg in 2012. For each field trial, the C6 and C3 phosphorylation of the 193 lines approximated a normal distribution. C6 and C3 phosphorylation were highly correlated in both years, with correlation coefficients *r* = 0.81 for 2011 and *r* = 0.86 for 2012. C6 and C3 phosphorylation results from the 2012 field trial are shown in Figure 1. The adjusted means produced by the first and second field trials were moderately correlated with *r* = 0.72 for C6 and *r* = 0.62 for C3, as shown in Figure 2. The proportion of the phosphorylation occurring at the C6 position was 69–82% in 2011 and 80–87% in 2012.

Climate data for Lincoln for the period 1 November to 30 March revealed a mean temperature of 16°C for the 2011 growing season and 14.5°C for 2012. The mean temperature for each month of the 2011 growing season was higher than the corresponding month of the 2012 season.

### Population structure

**Figure 3:**
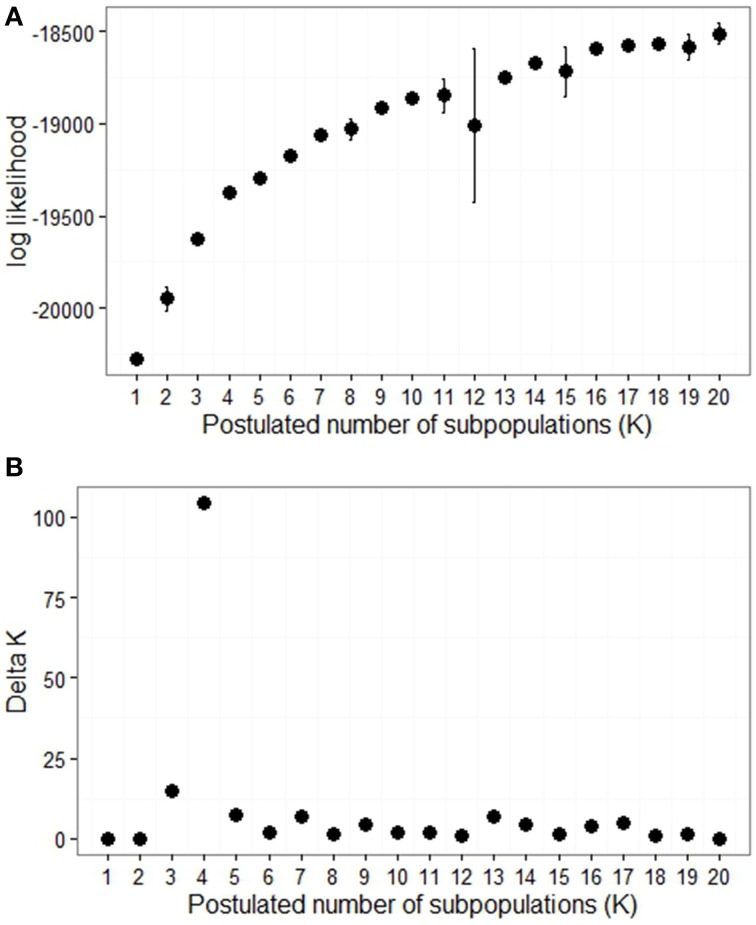
**Determination of population structure. (A)** The mean log likelihood of K (postulated number of subpopulations) for *K* = 1 to 20, with bars representing ± standard deviation. **(B)** Delta K for 1 to 20 subpopulations as calculated by Evanno et al. (2005).

SSR allele size and dosage were scored for 21 loci which spanned all 12 potato chromosomes. There were between three and 17 alleles per locus giving a total of 173 alleles. Analysis of the SSR genotype data using STRUCTURE software revealed a complex population structure. There was no clearly defined number of sub-populations, as indicated by the continual increase in log likelihood Ln P(D) (Figure 3A). However the slope of the graph decreases markedly after *K* = 4, indicating that four subpopulations may be the most useful estimate to use. The Delta K method, which targets a change in slope combined with low variance (Evanno et al., 2005), also indicated four subpopulations to be the best representation of the population structure (Figure 3B).

The four subpopulations indicated by STRUCTURE reflect the geographical origins of the samples (Supplementary Data 1) to some extent. For example, group 1 includes many old lines of unknown pedigree, including some which are thought to have been among the first potatoes brought to and grown in New Zealand. Most of the lines from North and South America fell into the largest group, group 2. Many of the lines from the UK were in group 3, whereas those from The Netherlands and Germany tended to be in group 4.

**Figure 4:**
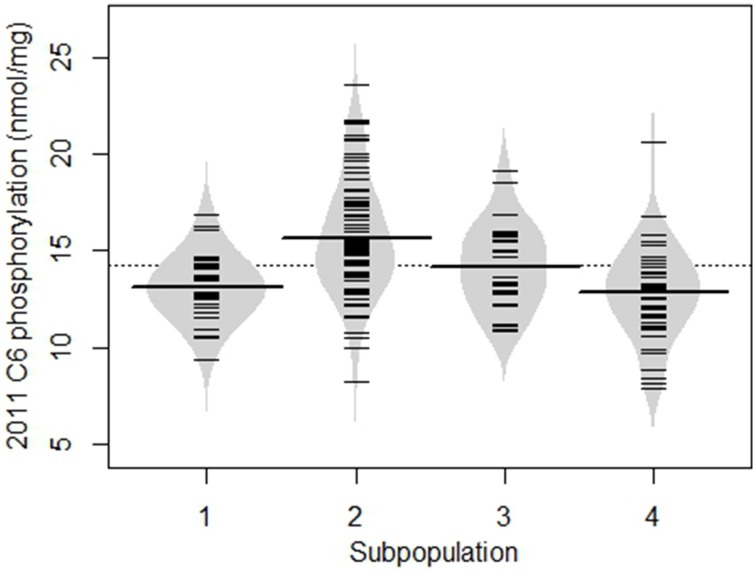
**Beanplot showing the degree of C6 phosphorylation in samples from the 2011 field trial, divided into the four subpopulations determined from SSR data using STRUCTURE**. The dotted line represents the overall mean; the long lines across each bean are mean values for that subpopulation, whereas the short lines represent individual potato lines. Similar results were obtained for the C6 2012, C3 2011, and C3 2012 datasets.

A relationship was evident between subpopulation membership and level of phosphorylation as shown for 2011 C6 phosphorylation in Figure 4. Most of the potato lines that had a high level of starch phosphorylation were in group 2. In addition, group 2 had the highest mean C6 phosphorylation. The same relationship was evident for C6 phosphorylation in 2012 and for C3 phosphorylation in both years.

### Genotyping candidate genes

Short amplicons (300–700 bp) from within the candidate genes of 192 potato lines were sequenced in both directions. This included two amplicons for *SSII*, none from *GBSS*, and one for each of the other six candidate genes. Analysis of the regions of the sequences which could be reliably determined in both the forward and reverse directions yielded 104 SNPs. The amplicon sizes and number of SNPs per amplicon are shown in Table 1. Four SSR markers were used for candidate gene genotyping, two from the *GBSS* gene which each had 11 alleles, one from *SBEII* with 12 alleles and one from *GWD* with five alleles. An insertion/deletion in the *GBSS* gene was amplified producing products of 60 and 200 bp, which were easily distinguished on an agarose gel. Image analysis software was effective for determining allele dosage in the heterozygotes. A list of the DNA polymorphisms and their positions on reference genes is given in Supplementary Data 4.

**Table 1:** **Summary of genotyping methods used for each candidate gene**.

**Candidate gene (GenBank accession No.)**	**Size of amplicon(s) sequenced (bp)**	**Number of SNPs**	**Other markers**
*SSI* (Y10416)	315	5	
*SSII* (X87988)	327, 302	11, 6	
*SSIII* (X94400)	587	14	
*SBEI* (X69805)	600	8	
*SBEII* (AJ000004)	442	12	SSR 12 alleles
*GWD* (Y09533)	690	32	SSR 5 alleles
*PWD* (GU045560)	523	16	
*GBSS* (X58453)			SSR (STGBSS) 11 alleles SSR (STWAX-2) 11 alleles InDel 140 bp

### Association analysis

**Figure 5:**
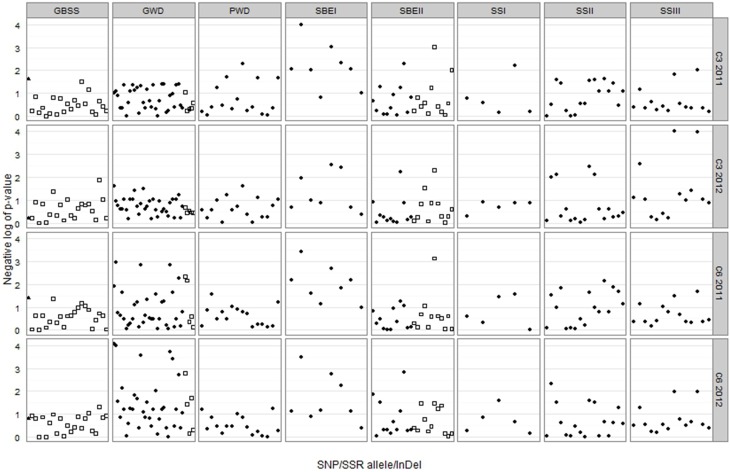
**Trellis plot of the negative log of *p*-value for each combination of genetic marker and trait-year**. The *GBSS* InDel is represented by a triangle, SNPs are represented by circles and SSR alleles by squares. A negative log value of 3 corresponds to *p* = 0.001, and a negative log of 2 corresponds to *p* = 0.01.

DNA polymorphisms were tested individually for association with the C6 and C3 phosphorylation adjusted means for each of the two field trials, the results of which are shown in Figure 5. SNPs in three genes gave association at *p* < 0.001 as shown in Table 2. Five of these SNPs were in the *GWD* gene and were associated with C6 phosphorylation (*p* < 0.001) for the 2012 samples, and of these, three also gave association at *p* < 0.01 for the 2011 samples. Two SNPs in the *SBEI* gene were associated with both C6 and C3 phosphorylation. Sbei2ag was associated (*p* < 0.001) with C6 for both years and C3 2011. Sbei5tc was associated with C3 2011 at *p* < 0.001 and also with C3 2012 and C6 for both years at *p* < 0.01. Two SNPs in the *SSIII* gene were associated with C3 phosphorylation at *p* < 0.001. Both ssiii7tc and ssiii11gc were associated with C3 phosphorylation in 2012 (*p* < 0.001) while ssiii11gc was also associated with C3 2011 at a lower level of significance (*p* < 0.01). Only one of the SSR alleles (sbeii180) gave association results significant at *p* < 0.001. It was associated with both C6 and C3 phosphorylation in 2011(*p* < 0.001) and also with C3 in 2012 (*p* < 0.01). In total, 9 SNPs and one SSR allele gave association results significant at *p* < 0.001 (Table 2), and another 20 SNPs/SSRs at 0.001<p < 0.01 (Supplementary Data 5). The *GBSS* insertion/deletion marker was not significantly associated with either phosphorylation trait.

**Table 2:** **SNP/SSR and trait combinations associated at *p* < 0.001**.

**SNP/SSR**	**Trait/year**	***p*-value**	***q*-value**	**Other trait/year combinations significant at *p* < 0.01**	**Minor allele frequency**	**Allele substitution effect (nmol/mg)**	**Allele associated with high phosphorylation**
gwd1ct	C6 2012	0.00008	0.0057		0.422	1.42	T
gwd2gt	C6 2012	0.00010	0.0057	C6 2011	0.038	3.68	T
gwd14ga	C6 2012	0.00027	0.0102	C6 2011	0.040	3.21	A
gwd26ag	C6 2012	0.00020	0.0057	C6 2011	0.040	3.09	G
gwd27cg	C6 2012	0.00037	0.0105		0.456	1.28	C
sbei 2ag	C3 2011	0.00010	0.0057		0.475	0.21	A
sbei 2ag	C6 2011	0.00039	0.0105		0.475	0.85	A
sbei 2ag	C6 2012	0.00033	0.0105		0.475	1.24	A
sbei5tc	C3 2011	0.00098	0.0202	C3 2012, C6 2011, C6 2012	0.111	0.35	T
ssiii7tc	C3 2012	0.00010	0.0057		0.268	0.32	T
ssiii11gc	C3 2012	0.00011	0.0057	C3 2011	0.273	0.31	G
sbeii180	C3 2011	0.00098	0.0202	C3 2012	0.004	1.45	180
sbeii180	C6 2011	0.00074	0.0180		0.004	3.89	180

Testing 144 genetic markers against four traits involves 576 tests so a level of significance of *p* < 0.01 is likely to produce several false positives. Therefore, the use of 0.001 as a level of significance is more appropriate but may be too stringent and exclude some important positive results. To explore this issue further, *q*-values for the association tests were calculated, where q represents the proportion of the significant tests which are likely to be false positives (Storey and Tibshirani, 2003). A significance threshold of *p* < 0.01, which generated 48 associations, was equivalent to a *q*-value of 0.05 such that two to three of the 48 would be false positives. However, at *p* < 0.001, which gave 13 significant results, the *q*- value was 0.02 such that less than one result (0.26) is likely to be a false positive. Therefore, *p* < 0.001 makes a sensible threshold to use for confidence that the genetic markers discovered are truly associated with the phenotype, and that we have got the most important markers. Although this is likely to exclude some true positives, some of those excluded will involve SNPs that have been found to be associated at *p* < 0.001 with the alternative form of phosphorylation or for the alternative year, e.g., SNP gwd2gt was significant at *p* < 0.001 for C6 2012 and 0.001<p < 0.01 with C6 2011 (Supplementary Data 5).

**Figure 6:**
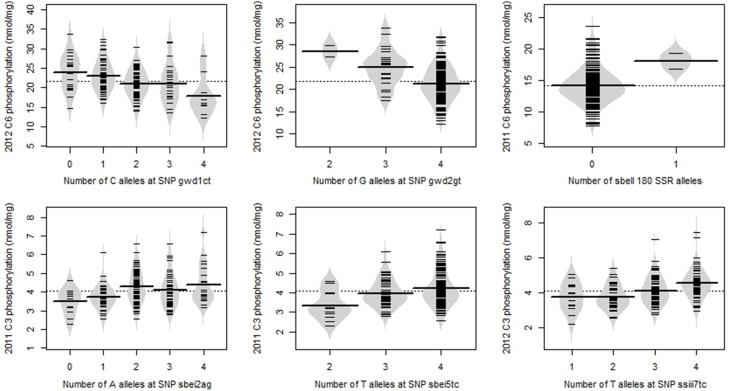
**Beanplots showing the relationship between allele copy number and degree of phosphorylation for representative examples of the most significant SNP/SSRs**. The overall mean is shown as a dotted line; the mean value for each allele copy number is shown as a line across the middle of each bean, and results for individual potato lines are shown by the short lines within the beans.

Of the 9 SNPs significantly associated with phosphorylation at *p* < 0.001, three (gwd1ct, gwd27cg, sbei2ag) had two alleles with fairly even frequency (Table 2), such that all five possible genotypes were observed. The minor allele frequencies of the other six SNPs were lower (0.038–0.273) such that only three or four of the five possible genotypes were observed. The SSR allele sbeii180 had a very low frequency (0.004), and only two genotypes were observed, with just three samples carrying the allele. The relationship between allele copy number and degree of phosphorylation for representative examples of the significant SNPs are shown in Figure 6. Where the graphs for two or more of the significant genetic marker-trait combinations were very similar, the example with the lowest *p*-value is shown.

The SNPs gwd2gt, gwd14ga, and gwd26ag gave very similar results, with identical allele dosage for all but three lines, and therefore appear to represent two haplotypes. They had similar allele frequencies and gave similar *p*-values. The very few differences between them may be due to errors in determining the genotypes or new rare mutations within the haplotypes. Similarly, SNPs ssiii7tc and ssiii11gc gave identical results in all but four lines, and may likewise represent two haplotypes.

Of the 9 SNPs which were significant at *p* < 0.001, one (ssiii7tc) was located within an intron, while two (gwd1ct, gwd26ag) were within synonymous codons, and would therefore be unlikely to affect protein function. The other six SNPs would result in amino acid substitutions in the protein encoded. These substitutions were analyzed using PROVEAN software (Choi et al., 2012) to predict whether an amino acid substitution would affect the biological function of a protein, by referring to the variation observed in homologous sequences. Using the default threshold of −2.5, the amino acid substitutions derived from the SNPs gwd2gt, gwd14ga, gwd27cg, sbei5tc, and ssiii11gc were considered to be neutral changes. However, the serine/asparagine substitution encoded by sbei2ag gave a “deleterious” result (−2.95), indicating that the change is likely to affect protein function.

The *SBEII* SSR has repeats of the codon GAA, which generates variation in the number of glutamic acid residues near the C-terminus of the protein. One of the 12 alleles observed using the *SBEII* SSR marker, sbeii180, was significant at *p* < 0.001 even though the allele only occurred in three potato lines. The *SBEII* SSR alleles ranged in size from 165 to 201 bp, indicating that considerable variation is tolerated in this region of the protein, such that the sbeii180 allele is unlikely to affect biological function.

## Discussion

### Phenotyping

The range of C6 phosphorylation of potato starch reported here (8–34 nmol/mg) is consistent with published results, such as 8–25 nmol/mg (Bay-Smidt et al., 1994) and 14–33 nmol/mg (Blennow et al., 1998). The larger range of values found here is likely due to the larger number of potato lines included. The C6 phosphorylation was higher in the 2012 field trial than in 2011, possibly due to different climatic conditions. The mean temperature during growth of the 2011 field trial was 1.5°C higher than in 2012. Growth temperature has been shown to affect the degree of phosphorylation in potato starch, with a high growth temperature resulting in decreased starch phosphorylation (Tester et al., 1999). The proportion of the starch phosphorylation occurring at the C6 position was found here to have a range of 69–87%, while published values range from 60–80% (Bay-Smidt et al., 1994; Haebel et al., 2008; Carpenter et al., 2012). C3 phosphorylation of large numbers of potato samples has not previously been reported because the measurement of C3 phosphorylation is more difficult and time consuming than for C6 phosphorylation, and the majority of the phosphorylation is at the C6 position.

### Genotyping

Three different genotyping methods were used, using both previously published markers and markers developed in this study. Amplicon sequencing proved to be the most efficient method for genotyping as it yielded many SNPs per amplicon, providing more information relative to the time and cost required compared to SSRs and the InDel. Amplicon sequencing could be performed much more effectively now that methods have been developed using next generation sequencing with barcodes to label the samples. SSRs were used for the population structure work as there were plenty of published SSRs to choose from. The use of image analysis software proved to be effective for determining allele dosage for an InDel run in an agarose gel. However, this method would be practical only for InDels that can be resolved by agarose gel electrophoresis.

Estimating the allele dosage is not always done in genotyping of tetraploids (Baldwin et al., 2011; Urbany et al., 2011) but is required to provide full genotypic information (Pajerowska-Mukhtar et al., 2009). Homozygotes were generally easy to score for all the genotyping methods used, but heterozygotes were sometimes difficult. In most cases the DAx software (Van Mierlo Software Consultancy, Eindhoven, The Netherlands) gave the same results for the forward and reverse amplicon sequences, which inspired confidence in the SNP allele dosage scored. However, occasionally the two sequences differed, in which case a decision was made based on sequence quality. Similarly, in some cases scoring the allele dosage of SSRs based on peak area was straightforward whereas other SSRs proved to be more difficult, possibly due to null alleles. The approach used for scoring the SSR alleles undoubtedly fails to recognize some null alleles but we considered it to be better than simply scoring presence/absence of alleles, as when dosage is not recorded much information is lost. The value of incorporating allele dosage in the association model is illustrated by the relationship between allele dosage and degree of phosphorylation as shown in Figure 6.

### Population structure

The collection of potato lines used here had a detectable, but not unequivocal, population structure, as might be expected for a diverse collection of lines which included modern breeding lines and heritage cultivars. This population structure would be likely to have confounded the association analysis, had it not been addressed in the model which was fitted. The subpopulation designated group 2 tended to have higher C6 phosphorylation than the other groups, therefore alleles common in group 2 might have been erroneously associated with starch phosphorylation. Previous studies have found population structure in collections of potato lines: a worldwide collection of 221 commercial lines including ancient lines plus those bred for processing, starch and fresh use, comprised six subpopulations (D'hoop et al., 2010), whereas a collection of 184 breeding lines was made up of 15 subpopulations (Pajerowska-Mukhtar et al., 2009). More recently, a collection of 250 lines, mostly North American breeding lines but including some genetic stocks and wild species, consisted of four subpopulations, with “minimal substructure” within cultivated potato (Hirsch et al., 2013). However, other studies of collections of breeding lines revealed an absence of population structure (Li et al., 2008; Urbany et al., 2011). In cases where population structure was detected, the structure was described as “complex” (Pajerowska-Mukhtar et al., 2009) or “weak” (D'hoop et al., 2010) and subpopulations were not clearly defined, with considerable overlap occurring between the inferred subpopulations, consistent with the results reported here. This is likely due to the long and complex history of interbreeding which has been used to produce the world's commercial potato cultivars.

### Association analysis

The use of *q*-values confirmed that a significance threshold of *p* < 0.001 was appropriate to give confidence that the genetic markers deemed to be significantly associated with phosphorylation levels were correctly assigned and could therefore be effective as genetic markers for marker assisted selection, and also that the most important markers have been included as positive results. Using a candidate gene approach meant that we could expect many of the markers to be significantly associated, but as many genes may be involved in determining starch phosphorylation levels, most exerting only small effects, only the strongest effects would be detected. Phenotype data were collected from two field trials run in consecutive years, and analyzed separately, as a means of determining how reproducible the results were. Although only one SNP (sbei2ag with C6 phosphorylation) gave results that were significant at *p* < 0.001 for both field trials, several of the other results that were significant at *p* < 0.001 were backed up by results from the alternative year that were significant at *p* < 0.01 (Table 2).

Of the 10 most significant SNP/SSR markers identified here, only one, SNP sbei2ag, appears likely to affect the starch phosphorylation phenotype directly by altering protein function. The other significant polymorphisms are therefore presumed to be in linkage disequilibrium with functional mutations, rather than affecting gene function directly, and may therefore be useful as genetic markers.

The *GWD* gene emerged as the most important gene associated with starch C6 phosphorylation, with five SNPs associated at *p* < 0.001 with C6 2012, three of which were also associated at *p* < 0.01 for C6 2011. This is consistent with the fact that *GWD* encodes the enzyme which catalyzes the C6 phosphorylation reaction. The SNPs gwd1ct and gwd2gt correspond to the A and H haplotypes previously reported to be associated with the degree of starch phosphorylation (Uitdewilligen, 2012). The combined results of two independent studies provide very strong evidence for this association. In addition, the *GWD* amplicon sequenced here overlaps a SCAR marker in the *GWD* gene, which has been shown to co-localize with a QTL for starch phosphorylation (Werij et al., 2012).

It is perhaps surprising that only a single, relatively weak (*p* = 0.005) association was found between *PWD* SNPs and C3 phosphorylation, given the *PWD* encodes the enzyme which phosphorylates starch at the C3 position. However, this may simply reflect that we were looking at SNPs from a single fragment of 532 bp and these SNPs were not in LD with polymorphisms that may affect C3 phosphorylation. Although we have not characterized the extent of LD in this association panel, other studies have indicated that LD decays rapidly over relatively short distances in tetraploid potato (Simko et al., 2006). Alternatively, naturally occurring allelic variation in the *PWD* gene affecting C3 phosphorylation may not occur in our collection of potato lines.

Polymorphisms in the genes encoding the starch branching enzymes *SBEI* and *SBEII* were significantly associated (*p* < 0.001) with the degree of both C6 and C3 phosphorylation. This is consistent with findings from antisense experiments that showed a decrease in expression of SBEI (Safford et al., 1998) or SBEII (Jobling et al., 1999) resulted in increased phosphorus content of potato starch. The sbei2ag SNP, which had a highly significant association with both C6 and C3 starch phosphorylation (C6 phosphorylation for both years, and C3 phosphorylation for 2011, at *p* < 0.001), encoded a serine/asparagine substitution at a position in the protein which tends to be conserved among homologous sequences, which have serine at this position. In potato the alleles encoding serine and asparagine are approximately equally common, and it is the asparagine variant which is associated with higher phosphorylation. It is possible that this SNP is a functional mutation which influences the degree of starch phosphorylation. Comparison with the crystal structure of the homologous protein in rice revealed that the equivalent serine residue is in the central catalytic α-amylase domain of the protein, but is not one of the seven residues known to be involved in enzymatic activity (Noguchi et al., 2011). However, there are other ways in which this substitution could affect starch phosphorylation, such as by altering protein/protein interactions. The *SBEII* SSR reported in this study produces variation in the number of glutamic acid residues near the C-terminal end of SBEII, an observation which has been reported previously (Jobling et al., 1999) but which has not been linked to functional variation in the protein. The sbeii180 allele which was associated with both C6 and C3 phosphorylation at *p* < 0.001 had a very low minor allele frequency of 0.004 and only occurred in three potato lines. It has been suggested that associations based on rare alleles may have an elevated false discovery rate compared to common alleles (Lam et al., 2007; Tabangin et al., 2009) but that it is preferable to treat such associations with caution rather than discard them (Moskvina et al., 2006; Tabangin et al., 2009) as rare alleles can be of interest. Therefore, the association of the sbeii180 allele with starch phosphorylation may require further validation. Incidentally, the three lines carrying the sbeii180 allele (Granola, V153 and V493) are not closely related, a fact which supports the validity of the association.

Among the four starch synthase genes that we examined, only *SSIII* had SNPs which were associated with starch phosphorylation at *p* < 0.001. Two SNPs in the *SSIII* gene were associated with C3 phosphorylation at *p* < 0.001, but none with C6 phosphorylation (*p* > 0.01). This is a novel observation as other studies have not measured C3 phosphorylation in a large number of samples. However, in a QTL analysis, a CAPS marker in *SSIII* was found to be associated with total starch phosphorylation (Werij et al., 2012). When C6 and C3 were combined to give total phosphorylation in this study, the association of the two *SSIII* SNPs was less significant, *p* = 0.004, than for C3 on its own (data not shown). The QTL analysis (Werij et al., 2012) also observed co-localization of *SSII* with a starch phosphorylation QTL, whereas our results yielded a relatively weak association with *SSII* SNPs (minimum *p* = 0.003). The 140 bp InDel in the *GBSS* gene has been shown to have a major effect on GBSS activity and amylose content (van de Wal et al., 2001) but in our study it did not significantly affect starch phosphorylation.

The markers that were associated with the greatest influence on C6 phosphorylation, and therefore on total phosphorylation, were in the *GWD, SBEI*, and *SBEII* genes. As some of these markers appear to be closely linked, only a subset would be required for use as genetic markers. The gwd1ct, gwd2gt, sbei2ag SNPs and the sbeii180 SSR allele, either individually or in combination, would make useful markers for potato starch phosphorylation and its accompanying physicochemical characteristics. The ssiii7tc or ssiii11gc SNPs may also be useful if C3 phosphorylation is of interest. While the C6 and C3 phosphorylation of starch have different roles in starch structure and catabolism (Hansen et al., 2009), it is not known if they differ in relation to their effects on the behavior of starch for industrial uses.

## Author contributions

MAC co-ordinated the project, contributed to genotyping and phenotyping of samples, analyzed data, and drafted the manuscript; NIJ did the mass spectrometry; RAG provided potato tubers and planted and harvested the field trials, RDC contributed to starch isolation and genotyping; SRM contributed to genotyping; ADN conducted the association analysis; RCB designed the field trials and analyzed phenotypic data; GMT conceived and initiated the project, secured funding, and contributed to the manuscript. All authors read and approved the final manuscript.

### Conflict of interest statement

The authors declare that the research was conducted in the absence of any commercial or financial relationships that could be construed as a potential conflict of interest.
