# Supplementary material for: Starch phosphorylation in potato tubers is influenced by allelic variation in the genes encoding glucan water dikinase, starch branching enzymes I and II, and starch synthase III
Source: Front Plant Sci. 2015 Mar 10;6:143. doi: 10.3389/fpls.2015.00143 (PMC4354307; doi:10.3389/fpls.2015.00143)
Supplement: Supplementary file 1 [file DataSheet1.ZIP › Table 2.DOCX]

Supplementary data 2. Previously published SSR primers used in this study. STM primers were reported by Milbourne et al., 1998, STI primers by Feingold et al., 2005, and STGBSS and STWAX-2 by Ghislain et al., 2004.

| Primer set | Annealing temp  (°C) | Mg concentration  (mM) | Product size  (bp) | Chromosome |
| --- | --- | --- | --- | --- |
| STI 04 | 55 | 3.5 | 98-124 | VI |
| STI 07 | 55 | 2.5 | 139-155 | XII |
| STI 09 | 58 | 2.5 | 277-304 | I |
| STI 13 | 60 | 2.5 | 269-321 | III |
| STI 20 | 55 | 3.5 | 126-140 | IV |
| STI 23 | 55 | 2.5 | 184-216 | X |
| STI 51 | 55 | 4.0 | 167-203 | XII |
| STI 55 | 60 | 2.5 | 234-260 | IV |
| STI 61 | 55 | 2.5 | 148-154 | III |
| STM 1041 | 53 | 2.5 | 110-118 | V |
| STM 1057 | 50 | 2.5 | 124-135 | VIII |
| STM 2005 | 50 | 2.5 | 169-213 | XI |
| STM 2012 | 60 | 3.5 | 258-277 | X |
| STM 2013 | 60 | 2.5 | 164-189 | VII |
| STM 2022 | 58 | 2.5 | 197-253 | II |
| STM 3009 | 58 | 2.5 | 163-188 | VII |
| STM 3012 | 50 | 2.5 | 183-218 | IX |
| STM 3018 | 58 | 2.5 | 178-190 | II |
| STM 5130 | 55 | 2.5 | 272-286 | XI |
| STM 5136 | 55 | 3.0 | 233-265 | I |
| STM 5148 | 55 | 3.0 | 423-495 | V |
| STGBSS | 53 | 2.5 | 146-158 | VIII |
| STWAX-2 | 53 | 2.5 | 237-256 | VIII |
