# Supplementary material for: Starch phosphorylation in potato tubers is influenced by allelic variation in the genes encoding glucan water dikinase, starch branching enzymes I and II, and starch synthase III
Source: Front Plant Sci. 2015 Mar 10;6:143. doi: 10.3389/fpls.2015.00143 (PMC4354307; doi:10.3389/fpls.2015.00143)
Supplement: Supplementary file 1 [file DataSheet1.ZIP › Table 3.DOCX]

Supplementary data 3. PCR primers developed and used in this study

| Primer name | Primer sequence | Product size | Gene |
| --- | --- | --- | --- |
| Primers for whole gene sequencing | | | |
| SSI2206f  SSI2206r | ttgaaagattttgtctttacatgattc  tccactcttctcccaaagga | 9 kb | *SSI* |
| SSII7201f  SSII2633r | gtaatgcgctgctcaatgaa  atgatcaagtgcggcaatc | 7 kb | *SSII* |
| SSIII4128f  SSIII4128r | aaaccccattaaagcaaatacg  ttttgcatcctttgatttctca | 15 kb | *SSIII* |
| GBSS4422f  GBSS4422r | ggctgttgacagagtaatcagg  ttagcctgccagcttctttc | 4 kb | *GBSS* |
| SBEI7850f  SBEI7850r | agattgctccaacatattttcc  cctcgatattggctgttggt | 8 kb | *SBEI* |
| SBEII8967f  SBEII8967r | catgcgacccatcacaataa  tggcagaaaaatctcccaga | 9 kb | *SBEII* |
| SBEII11253f  SBEII11253r | cctcatgggtccagagtgaa  gccaccaaaaagtggatcat | 11 kb | *SBEII* |
| GWD15213f  St010 | ttgcaaaaacatccaatcaaa  ggattgtctggcggaagtta | 15 kb | *GWD* |
| PWD10087f  PWD10087r | atggtactgccagaccttgg  ggtgcaatttcagcctcaat | 10 kb | *PWD* |
| Primers for SSR markers | | | |
| SBEIIf  SBEIIr2 | gacgttgtaaaacgacggcctgatgatcgtcctcgttcaa  ttcaacgcgatcacaagttc | 165-192 bp | *SBEII* |
| St011  St012 | gacgttgtaaaacgacggcctacgtgatccaaagccatca  tgcaatacataatgcgtgtgt | 199-206 bp | *GWD* |
| Primers for amplicon sequencing | | | |
| SSI2206f  SSI 315r | ttgaaagattttgtctttacatgattc  ccaacaacagagccagaacc | 315 bp | *SSI* |
| SSIIseq1041f2  SSIIseq1041r | tccactaggtcactctggttcttgaaa  tgaagtaggtcctgttgcatggct | 345 bp | *SSII* |
| SSIIseq6311f2  SSIIseq6311r | cggctggtctaaagacagcagatcg  accgatcagtgggacatcatcacg | 302 bp | *SSII* |
| SSIIIseq587f  SSIIIseq639r | tggtaaaagccacgaagactcgtgat  accttagcacgcatagccgc | 587 bp | *SSIII* |
| SBEIseq600f  SBEIseq600r | accccgagccccacgaatct  acagcatcaacatctgtagcctcgc | 600 bp | *SBEI* |
| SBEII727f  SBEII474r  SBEII727fseq | agggagaagtctactttctttcacct  accaggtcaaatctccgtctgc  tgtatgctttattgatttagtttatcaag | 727 bp | *SBEII* |
| GWDseq690f  GWDseq690r | tgcaacttgagcttgagaaaggca  aaggtgtttcggcagccttgt | 690 bp | *GWD* |
| PWDseq532f  PWDseq532r | agtgaccaaggtgcaccagca  ccgttgggctcagtgtgtgga | 532 bp | *PWD* |
