# Supplementary material for: Starch phosphorylation in potato tubers is influenced by allelic variation in the genes encoding glucan water dikinase, starch branching enzymes I and II, and starch synthase III
Source: Front Plant Sci. 2015 Mar 10;6:143. doi: 10.3389/fpls.2015.00143 (PMC4354307; doi:10.3389/fpls.2015.00143)
Supplement: Supplementary file 1 [file DataSheet1.ZIP › Table 4.DOCX]

Polymorphisms are shown on the potato DM reference genome from the Potato Genome Sequencing Consortium database [http://potatogenomics.plantbiology.msu.edu](http://potatogenomics.plantbiology.msu.edu/). Primer binding sites are underlined. SSRs are shown in bold. SNPs were numbered in 5’ to 3’ order except where indicated otherwise.

SSI: PGSC0003DMG402018552 (8805-8491)

TTGAAAGATTTTGTCTTTACATGATTCTTGATTTTACAGCAGGTGTCAATACCAAATGGGGTCTCTGCAAACACCCACAAATCTTAGCAATAAGTCATGTTTATGTGTGTC[A/G]GGGAGAGTTGTGA[G/A]GGGTTTGAGGGTAGAAAGACAAGTGGGGTTGGG[A/G]TTTTCTTGGTTGTTGAAGGGACGAAGAAACAGAAA[G/A]GTTCAATCTTTGTGTGTTACAAGTAGTG[T/C]TTCAGATGGTTCATCAATTGCTGAAAATAAGAAAGTGTCAGAAGGGCTTCTTTTGGGTGCTGAGAGAGATGGTTCTGGCTCTGTTGTTGG

SSII: PGSC0003DMG400001328 (6908-6582)

TCCACTAGGTCACTCTGGTTCTTGAAATCTTGGATTCCTATTATCCCTGTGAACTTCATCTTTTGTGATTTCTACTGTAATGGAGAATTCCATTCTT[C/G]TTCATAGTGGAAATCAGTTCCACCCCAACTTA[C/T]CCCTT[T/G]T[A/C]GCACTTAGGCCCAAAAAATTATCTCTAATTCAT[G/C]GCTCCAGTAGAGAG[C/A]AAATGTGGAGGATCAA[A/G]CGCGTTAAAGCAACA[G/C]GT[G/A]AAAATTCTGGG[G/A]AAGCTGCAAG[T/G]GCTGATGAATCGAATGATGCCTTACAGGTTACAATTGAAAAGAGCAAAAAGGTTTTAGCCATGCAACAGGACCTACTTCA

SNP 11ga is out of order; it lies between 8gc and 9ga.

SSII: PGSC0003DMG400001328 (1623-1322)

CGGCTGGTCTAAAGACAGCAGATCGTGTAGTTACAGTTAGTCATGGATATTCATGGGAACTAAAGACTTCCCAAGGTGGTTGGGGATTGCATCAGATAATTAATGAGAACGA[T/C]TGGAAATTAC[A/G]GGGTATTGTGAATGGGATTGATACAAAAGAGTGGAACCCTGAGTTGGA[C/T]GTTCACTTACAGTCAGATGGTTAC[A/G]TGAACTACTCCTTGGACAC[G/A]CT[A/G]CAGACTGGCAAGCCTCAATGTAAAGCTGCATTGCAGAAGGAACTTGGTTTACCAGTTCGTGATGATGTCCCACTGATCGGT

SSIII: chr02 (36365621-36366207)

TGGTAAAAGCCACGAAGACTCGTGATATCACCTGGTACATAGAGCCAAGTGAATTTAAATGCGAGGACAAGGTCAGGTTATACTATAACAAAAGTTCAGGTCCTCTCTCCCATGCTAAGGACTTGTGGATCCACGGAGGATATAA[T/C]AATTGGAAGGATGGTTTGTCTATTGT[C/T]AAAAAGCTTGTTA[A/G]ATCTGAGAGAATAGATGGTGATTGGTGGTATACAGAGGGT[T/A]A[G/A]TGGGGATG[T/C]TATTAT[T/C]ATACGTACTTGCACTTCTTTTAGTTAGTGCTTGCATGAGCTTCCACAATTATACTTTTGTCTGTCTCCTTCTATTTCT[T/C]CTAAATGTT[T/C]GGCTACACTCTTCTGAGGAATGTACTTTTCTGCAGTTGTTATTCCTGATC[A/G]GGCACTT[T/G]TCTTGGATTGGGTTTTTGCTGATGGTCCACCCAA[G/C]CATGCCATTGCTTATGATAACAATCACCGCCAAGACTTCCATGCCATTGTCCCCAA[C/G]CACATTC[C/T]GGAGGAATTATATTGGGTTGAGGAAGAACATCAGATCTTTAAGACACTTCAGGAGGAGAGAAGGCTTAGAGAAGCGGCTATGCGTGCTAAGGT

SNP 14ga is out of order; it comes between SNPs 4ta and 5tc.

SBEI: [PGSC0003DMG400009981](http://potato.plantbiology.msu.edu/cgi-bin/annotation_report.cgi?gene_id=PGSC0003DMG400009981) (4782-4183)

ACCCCGAGCCCCACGAATCTACGAAGCACATGTCGGCATGAGCAGCTCTGAGCCACGTGTAAATTCGTATCGTGAGTTTGCAGATGATGTTTTACCTCGGATTAAGGCAAATAACTATAATACTGTCCAGTTGATGGCCATAATGGAACATTCTTACTATG[G/C]ATCATTTGGATATCATGTTACAAACTTTTTTGCTGTGAGCA[A/G]TAGATATGGAAACCC[G/A]GAGGAC[C/T]TAAAGTATCTGATAGATAAAGCACATAGCTTGGGTTTACAGGTTCTGGTGGATGTAGTTCACAGTCATGCAAGCAATAATG[T/C]CACTGATGGCCTCAATGGCTTTGATATTGGCCAAGGTTCTCAAGAATCCTACTTTCATGCTGGAGAGC[G/A]AGGGTACCATAAGTTGTGGGATAGCAGGCTGTTCAACTATGCCAATTGGGAGGTTCTTCGTTTCCTTCT[T/G]TCCAACTTGAGGTGGTGGCTAGAAGAGTATAACTTTGACGGATTTCGATTTGATGGA[A/G]TAACTTCTATGCTGTATGTTCATCATGGAATCAATATGGGATTTACAGGAAACTATAATGAGTATTTCAGCGAGGCTACAGATGTTGATGCTGT

SBEII: chr09 (3734857-3734416)

TGTATGCTTTATTGATTTAGTTTATCAAGTTCTCATGTAATGCTTCACCCCCACCCCCAACTATCATTTTCAGGATATGTATGATTTTATGGCT[C/T]TGGATAGACC[G/A]TCAACA[T/C]CA[T/G]TAATAGATCGTGGGATAGCATTGCACAAGATGATTAGGCTTGTAACTATGGGATT[A/G]GGAGGAGAAGGGTACCTAAATTTCATGGGAAATGAATTCGGCCACCC[T/C]GGTATAGATTGCTAACCCTCCTT[G/T]G[C/T]CATATATCTTC[T/C]CTTGGCTGCTGTTGATCTT[G/A]TACAATTTTCTTTCCTGGTATTCTTT[A/T]T[A/G]GTACAGTAATACATTCATTAATGTTTTTCCAGAGTGGATTGATTTCCCTAGGGCTGAACAACACCTCTCTGATGGCTCAGTAATTCCCGGAAACCAATTCAGTTATGATAAATGCAGACGGAGATTTGACCTGGT

SNP 11tc is out of order; it lies between 8ct and 9ga.

GWD PGSC0003DMG400007677 (11485-10796)

TGCAACTTGAGCTTGAGAAAGGCATTACCCTTGATGAGTTGCGGAAAAAGATTACAAAAGGGGAGATAAAAACTAAGGCGGAAAAGCACGTGAAAAGAAG[C/T]TCTTTT[G/T]CCGTTGAAAGAATCCAAAGAAAGAAGAGAGACTTTGGGCA[G/T]CTTATTAATAAGTAT[A/C]CTTCCAGTCCTGCAGT[A/G]CAAGTACAAAAGGTCT[C/T]GGAAGAAC[A/C]A[C/G][C/T][A/T]GCCTTATCTAAAATTAAG[A/C]TGTATGCCAAGGAGAAGGAGGAGCAGATTGATGATCCGATCCT[A/T]AAT[A/G]AAAAGATCTTTAAGGTC[G/A]ATGATGGGGAGCTACT[G/T]GTGAGTGCTAACTTATTCTTGACTGTAACCACTAATCTCAGAAGC[A/T]AGTTTGA[A/G]TGCATATTGAGAATCTTCCATTGTCTCCACATTTTAAGGTA[C/G]TGGTA[G/T][A/C]AAAGTCCT[C/G]TGG[C/G]AAGACAAA[A/G]GTACAT[C/A/G]TAGCTACAGATCTGAATCA[G/T]CCAATTACTCTTCACTGGGCATT[A/G]TCCAAAAGTC[C/G]TGGAGAGTGGATGGTAAGAATAAAAATCGTGCACATCTTGCAATTGAAAAAACCAA[A/C]GAAGCAAG[A/G]TA[G/T]AAAACCTTCCTATACCCTACCTATATTGA[A/G]TA[A/G]TCAGTGACCATTTACATTTTCAGGTACCACCTTCAAGCATATTGCCTCCTGGATCAATTATTTTAGACAAGGCTGCCGAAACACCTT

PWD PGSC0003DMG400016613 (10658-11180)

AGTGACCAAGGTGCACCAGCATCATTTAACGTTCCTGCAGGAGCAGTTATTCCATTTGGTTCCATGGAAACGGCATTG[G/A]AAA[C/T]GAACAAGTTAATGGAGACCTTCACATTG[G/C]TTGTCGAACAGAT[A/T]GAAACAGCTGAAATTGATGGCGGTGAACTTGATAAACA[T/C]TGTGAGGATCTCCAGAAGTTAATATCT[T/A]CTCTATTGCCTGGACAAGATGTCATTGAAAG[C/G]TTGGGAGA[A/G]G[T/C][A/G]TTTCCCGGTAATGCACGTTTAATAGTGCGTTCAAGTGCTAATGT[C/T]GAGGACTTGGCGGG[G/A]ATGTCAGC[T/A]GC[T/C]GGACTTTATGATTCAATTCCTAATGTTAGCCCTTCAGATCC[G/A]ATAAGGTTTGGACATGCTGTAGCCCGTGTTTGGGCCTCGTT[G/A]TATACTAGAAGAGCAGTACTGAGCCGCAGAGCTGCTGGTGTGTCCCAGAAAGACGCTACAATGGCCGTGCTAGTGCAAGAAATGCTTTCACCAGATTTATCTTTCGTCCTCCACACACTGAGCCCAACGG

GWD SSR: PGSC0003DMG400007677 (316-133)

TACGTGATCCAAAGCCATCACATCATGTTCACCTTCAGCTATTGGAGGAGAAGTGAGAAGTAGGAATTGCAATATGAGGAATAATAAGAAAAACTTTGTGAAAGCTAAATTAGCTGGGTATGATATAGGGAGAAATGTGTAAACATTGTAC**TATATATA**GTATACACACGCATTATGTATTGCA

SBEII SSR: chr09 (3730143-3729995)

TGATGATCGTCCTCGTTCAATTATGGTGTATGCACCTAGTAGAACAGCAGTGGTCTATGC

ACTAGTAGACA**AAGAAGAAGAAGAAGAAGAAG**TAGCAGTAGTAGAAGAAGAACCCATTGA

AGAATGAACGAACTTGTGATCGCGTTGAA

GBSS: chr08 (56786181-56785488)

The GBSS InDel is located approximately 650 bp 5’ of the start codon, and the GBSS SSRs both lie between the InDel and the start codon. The start codon and SSRs are shown in bold.

GAACCATGCATCTCAATCTTAATACTAAAAAATGCAACAAAATTCTAGTGGAGGGACCAGTACCAGTACATTAGATATTATTTTTTATTACTATAATAATAATTTAACTAACACGAGACATAGGAATGTCAAGTGGTAGCGGTAGGAGGGAGTTGGTTTAGTTTTTTAGATACTAGGAGACAGAACCGGAGGGGCCCATTGCAAGGCCCAAGTTGAAGTCCAGCCGTGAATCAACAAAGAGAGGGCCCATAATACTGTTGATGAGCATTTCCCTATAATACAGTGTCCACAGTTGCCTTCCGCTAAGGGATAGCCACCCGCTATTCTCTTGACACGTGTCACTGAAACCTGCTACAAATAAGGCAGGCACCTCCTCATTCTCA**CACTCACTCACTCACTCACTCACT**CACACAGCTCAACAAGTGGTAACTTTTACTCATCTCCTCCAATTATTTCTGATTTCATGCATGTTTCCCTACATTCTATTATGAATCGTGTTATGGTGTATAAACGTTGTTTCATATCTCATCTCATCTATTCTGATTTTGATTCTCTTGCCTACTGTAATCGGTGATAAATGTGAATGCTTCCT**CTTCTTCTTCTTCTT**CTCAGAAATCAATTTCTGTTTTGTTTTTGTTCATCTGTAGCTTGGTAGATTCCCCTTTTTGTAGACCACACATCAC**ATG**GCAAGCAT
