# Supplementary material for: Starch phosphorylation in potato tubers is influenced by allelic variation in the genes encoding glucan water dikinase, starch branching enzymes I and II, and starch synthase III
Source: Front Plant Sci. 2015 Mar 10;6:143. doi: 10.3389/fpls.2015.00143 (PMC4354307; doi:10.3389/fpls.2015.00143)
Supplement: Supplementary file 1 [file DataSheet1.ZIP › Table 5.DOCX]

Supplementary data 5. SNPs/SSRs significant at P<0.01. Red shading indicates P<0.001; yellow shading indicates 0.001<P<0.01.

| **SNP/SSR** | **C3 2011** | **C3 2012** | **C6 2011** | **C6 2012** |
| --- | --- | --- | --- | --- |
| gwd1ct | 0.100832 | 0.025018 | 0.012763 | 8.20E-05 |
| gwd2gt | 0.080692 | 0.108769 | 0.001122 | 0.000104 |
| gwd5ag | 0.441118 | 0.237832 | 0.022373 | 0.0072 |
| gwd14ga | 0.047769 | 0.145376 | 0.001401 | 0.000268 |
| gwd21cg | 0.498033 | 0.272434 | 0.027448 | 0.009798 |
| gwd26ag | 0.127449 | 0.133789 | 0.001422 | 0.000198 |
| gwd27cg | 0.107612 | 0.090583 | 0.023343 | 0.000373 |
| gwd30gt | 0.04012 | 0.06063 | 0.005269 | 0.001887 |
| gwd198 | 0.09191 | 0.204598 | 0.004436 | 0.001591 |
| gwd200 | 0.58648 | 0.35574 | 0.006884 | 0.035782 |
| pwd9tc | 0.005309 | 0.023214 | 0.166594 | 0.141472 |
| sbei1gc | 0.009099 | 0.208381 | 0.006359 | 0.073184 |
| sbei2ag | 9.75E-05 | 0.010571 | 0.000391 | 0.000332 |
| sbei3ga | 0.009408 | 0.097635 | 0.02568 | 0.125729 |
| sbei5tc | 0.00098 | 0.0029 | 0.002137 | 0.001787 |
| sbei6ga | 0.004635 | 0.003728 | 0.015148 | 0.00555 |
| sbei7tg | 0.009099 | 0.208381 | 0.006359 | 0.073184 |
| sbeii9ga | 0.005068 | 0.132589 | 0.089364 | 0.001523 |
| sbeii11tc | 0.057722 | 0.005954 | 0.054386 | 0.071806 |
| sbeii180 | 0.000975 | 0.004867 | 0.000739 | 0.034461 |
| sbeii201 | 0.00963 | 0.252574 | 0.899564 | 0.696017 |
| ssi4ga | 0.006187 | 0.131842 | 0.027082 | 0.225706 |
| ssii10412ct | 0.300989 | 0.010158 | 0.031035 | 0.004925 |
| ssii10413tg | 0.02682 | 0.007915 | 0.101192 | 0.03191 |
| ssii104110tg | 0.027461 | 0.003352 | 0.02328 | 0.02498 |
| ssii104111ga | 0.02682 | 0.007915 | 0.101192 | 0.03191 |
| ssii63112ag | 0.022983 | 0.652564 | 0.007416 | 0.210043 |
| ssiii2ct | 0.069791 | 0.002531 | 0.070942 | 0.052242 |
| ssiii7tc | 0.015126 | 9.74E-05 | 0.031219 | 0.010381 |
| ssiii11gc | 0.009535 | 0.000105 | 0.020536 | 0.010537 |
